# Intensified conditioning regimens with total marrow irradiation/etoposide/cyclophosphamide and busulfan/etoposide/cyclophosphamide overcome the impact of pre‐transplant minimal residual disease on outcomes in high‐risk acute lymphoblastic leukemia patients in complete remission

**DOI:** 10.1002/cam4.6897

**Published:** 2024-01-02

**Authors:** Xiaoyan Zhao, Ziwei Xu, Ziying Li, Xi Zhou, Yu Hu, Huafang Wang

**Affiliations:** ^1^ Department of Hematology, Union Hospital, Tongji Medical College Huazhong University of Science and Technology Wuhan China; ^2^ Department of Pediatrics, Union Hospital, Tongji Medical College Huazhong University of Science and Technology Wuhan China; ^3^ Department of Pathology, Union Hospital, Tongji Medical College Huazhong University of Science and Technology Wuhan China

**Keywords:** acute lymphoblastic leukemia, hematopoietic stem cell transplantation, intensified conditioning regimen, minimal residual disease

## Abstract

**Purpose:**

Among high‐risk acute lymphoblastic leukemia (ALL) patients undergoing allogeneic hematopoietic stem cell transplantation (allo‐HSCT), those with positive minimal residual disease (MRD) are susceptible to poor outcomes. Therefore, it is necessary to determine the most suitable preparatory regimen for these patients.

**Methods:**

Data were analyzed from 141 patients who received allo‐HSCT and were diagnosed with high‐risk ALL. These patients underwent intensified conditioning regimens, including either total marrow and lymphoid irradiation (TMLI)‐etoposide (VP16)‐cyclophosphamide (CY) or busulfan (BU)‐VP16‐CY between October 2016 and November 2022. A total of 141 individuals were in complete remission (CR) before transplantation and, among all patients, 90 individuals exhibited a negative MRD status and 51 patients had a positive MRD status.

**Results:**

In patients who tested negative for MRD, the incidence of relapse within a 2‐year timeframe was 25.0% (24.8%–25.5%), compared with 32.2% (31.2%–33.2%) in MRD‐positive patients; however, this difference was not statistically significant. There were no significant differences in the 2‐year disease‐free survival (DFS) and 2‐year overall survival (OS) rates between the MRD‐negative and MRD‐positive groups (DFS: 67.2% (57.9%–78.1%) vs. 55.5% (42.6%–72.3%); OS: 69.0% (61.9%–88.2%) vs. 66.7% (53.9%–82.5%)). Furthermore, no notable variations were observed in the occurrence of transplant‐related mortality (TRM) and graft‐versus‐host disease (GVHD) across the two groups.

**Conclusion:**

This study reveals the benefits of TMLI‐VP16‐CY and BU‐VP16‐CY conditioning regimens in high‐risk ALL patients with CR and MRD‐positive status. A large‐scale prospective clinical trial is warranted in the future.

## 1 INTRODUCTION

Allogeneic hematopoietic stem cell transplantation (allo‐HSCT) is an effective and potentially curative treatment method used for individuals with high‐risk acute lymphoblastic leukemia (ALL).^1^ However, patient prognoses following allo‐HSCT are highly variable.^2^, ^3^ For high‐risk ALL patients, a major predictor of clinical outcomes is pre‐transplant disease status, including minimum residual disease (MRD) status.^4^ Therefore, for high‐risk ALL patients receiving allo‐HSCT, it is necessary to accurately identify the disease status before transplantation and select the most appropriate transplantation regimen.

MRD positivity prior to transplantation has emerged as a notable risk factor associated with increased incidence of relapse in ALL patients receiving allo‐HSCT.^5^, ^6^ Currently, the presence of MRD cells before transplantation is mainly identified by flow cytometry, quantitative real‐time polymerase chain reaction (RQ‐PCR), and next‐generation sequencing.^7^, ^8^ Patients with persistent MRD before allo‐HSCT typically exhibit inferior outcomes compared with individuals who are MRD‐negative. One study showed that the adult ALL patients with MRD‐positive had a shorter 2‐year progression‐free survival compared with those with MRD‐negative (28% vs. 47%), and this difference was close to statistical difference.^9^ Moreover, another study also showed that MRD‐positive ALL patients experienced a higher relapse rate than MRD‐negative patients (30% vs. 16%).^10^ Hence, there is an urgent need to identify strategies to overcome the poor outcomes associated with pre‐transplant positive MRD in patients receiving allo‐HSCT. Therefore, it is critical to develop a better transplantation regimen for MRD‐positive patients.

The conditioning regimen constitutes a significant component of the treatment protocol, and may be modified to benefit MRD‐positive patients. Studies have shown that the primary therapeutic approach for patients with ALL undergoing allo‐HSCT entails the administration of a conditioning regimen that primarily focuses on total body irradiation (TBI) and cyclophosphamide (CY).^4^, ^11^ However, TBI leads to significant long‐term and short‐term radiation‐related toxicity,^12^ and has been positively correlated with a higher rate of transplantation‐related mortality (TRM).^13^ In recent years, to reduce radiation‐related toxicity, regimens have undergone successive revisions involving the implementation of total marrow and lymphoid irradiation (TMLI) and busulfan (BU). TMLI is a new treatment method that can more accurately eliminate tumor cells and reduce radiation‐related toxicity for patients receiving transplantation.^14^, ^15^ Additionally, increasing the intensity of the preparatory regimen for transplantation can influence clinical outcomes in high‐risk ALL patients. Studies from our center noted that the addition of idarubicin (IDA) to the TBI‐CY regimen significantly reduced the frequency of relapse and extended survival among high‐risk ALL patients.^4^, ^16^ These promising results may be related to the more complete clearance of leukemia cells by the intensive conditioning regimen. However, IDA has been gradually replaced by etoposide (VP16) due to mucosal toxicities and cardiotoxicity.^17^, ^18^ Importantly, it remains unclear whether the intensified conditioning regimens involving TMLI‐VP16‐CY and BU‐V16‐CY are able to significantly improve outcomes among high‐risk ALL individuals with MRD positivity.

Herein, we aimed to determine whether the intensified conditioning regimens involving TMLI‐VP16‐CY or BU‐VP16‐CY can improve outcomes among high‐risk ALL patients exhibiting MRD positivity at transplantation, to achieve similar outcomes as MRD‐negative patients.

## 2 MATERIALS AND METHODS

### 2.1 Eligibility criteria

From October 2016 to November 2022, a total of 141 patients with high‐risk ALL and undergoing allo‐HSCT at our transplantation facility were included in this study. The classification of high‐risk patients was defined according to previously published criteria.^3^, ^4^, ^19^ A cohort including 61 individuals who received a diagnosis of high‐risk ALL were subjected to the TMLI‐VP16‐CY conditioning regimen, while an additional 80 high‐risk ALL patients underwent the BU‐VP16‐CY conditioning regimen. Across the entire patient cohort, bone marrow morphology and cytology analyses, along with MRD assessment by both flow cytometry and RQ‐PCR, were carried out within 14 days prior to transplantation. The present study was approved by the Medical Ethics Committee of Huazhong University of Science and Technology and adhered to the principles outlined in the Declaration of Helsinki. In our clinical observations, the effects of non‐radiotherapy regimens for extramedullary infiltration are very limited. Consequently, the TMLI‐VP16‐CY conditioning regimen was chosen for high‐risk patients if they presented with extramedullary infiltration with significant involvement of the testis, liver, lymph node chains, brain, or spleen at the point of diagnosis or during the induction chemotherapy phase, or if extramedullary residual lesions were present prior to transplantation. For other high‐risk ALL patients without extramedullary infiltration throughout the disease course, the BU‐VP16‐CY conditioning regimen was used. Individuals who underwent the TMLI‐VP16‐CY conditioning regimen were categorized into the “TMLI‐VP16‐CY” group, while those who received the BU‐VP16‐CY conditioning regimen were categorized into the “BU‐VP16‐CY” group. Subsequently, within each of the aforementioned two groups, individuals with high‐risk ALL who underwent allo‐HSCT were further categorized into two distinct groups based on their disease status before transplantation. These groups included: (i) the “MRD‐negative” group, encompassing patients who achieved complete remission (CR) and displayed no indications of MRD during transplantation; (ii) the “MRD‐positive” group, encompassing patients who achieved CR but displayed any level of positive MRD detected by flow cytometry or RQ‐PCR in bone marrow mononuclear cells during transplantation.

### 2.2 Assessment of minimal residual disease

In our transplantation center, eight‐color flow cytometry and RQ‐PCR were used to determine MRD status. For flow cytometry, aberrant cells were defined according to published criteria.^20^, ^21^ Any level of disease residue was defined as MRD positivity. The sensitivity of MRD detection using the flow cytometry‐based method reached a threshold of 10^−4^. In addition to flow cytometry, RQ‐PCR based on fusion gene expression was also used to assess MRD. MRD positivity was characterized by the presence of fusion genes at any level. The sensitivity of MRD detection using the RQ‐PCR method was 10^−5^.

### 2.3 Conditioning regimens

For individuals with high‐risk ALL, either the TMLI‐VP16‐CY conditioning regimen or the BU‐VP16‐CY conditioning regimen was used. VP16 was added as intensification therapy. The TMLI‐VP16‐CY conditioning regime began with bone marrow irradiation on Day −8 using Tomo Therapy with a dosage of 8 Gy administered in two fractions. Irradiated regions included the skull (excluding the mandible), scapula and clavicle, sternum and rib cage, upper limbs, C1‐L5 spine, pelvic bones, and lower limbs. Apart from bone marrow irradiation, additional targeted areas including the brain, testis, spleen, major lymph node chains, and liver, which received irradiation at doses of either 10 or 8 Gy. Specifically, individuals who had extramedullary invasion during the period of diagnosis or as the disease progressed, but were in remission prior to transplantation, received prophylactic irradiation of 8 Gy, given in two fractions. Conversely, those with extramedullary residue before transplantation received 10 Gy irradiation (also in 2 fractions). For the TMLI‐VP16‐CY regimen, intravenous administration of VP16 (125 mg/m^2^/12 h) occurred on Days −5 and −4. Subsequently, CY (60 mg/kg/day) was administered intravenously for a duration of more than 2 h on Days −3 and −2. For the BU‐VP16‐CY regimen, intravenous administration of BU (3.2 mg/kg/day) took place from Days −9 to −7 for a duration exceeding 3 h on three consecutive days. Next, intravenous VP16 was delivered at a dosage of 5 mg/kg/12 h from Days −6 to −4. Finally, CY (at a dose of 60 mg/kg/day) was intravenously administered for a period of 2 h on Days −3 and −2.

### 2.4 Stem cell source

To facilitate the mobilization of stem cells from both peripheral blood (PB) and bone marrow (BM), the donors received a daily subcutaneous injection of recombinant human granulocyte colony‐stimulating factor at a dose that varied between 8 and 10 μg/kg every day. Harvested stem cells from bone marrow and peripheral blood were co‐transfused into recipients during HLA‐5/10‐matched haploidentical HSCT. For other HSCT settings, only the PBSCs were infused into patients. In our transplantation procedure, regardless of whether they were derived from donor BM or PB, the collected cells were infused into patients without any manipulation.

### 2.5 Assessment of engraftment and regimen‐related toxicities

The hematopoietic engraftment was assessed according to published methods.^3^ At 30, 90, 180, 270, and 360 days after the patients received HSCT, PCR capillary electrophoresis was used to evaluate chimerism. Any regimen‐related toxicity was assessed in line with Bearman's Grading System.^22^

### 2.6 Graft‐versus‐host disease (GVHD) prophylaxis

In the context of HLA‐matched sibling allo‐HSCT, a therapeutic protocol was implemented consisting of 5 mg/kg of cyclosporine A (CsA) (administered twice daily on Day −1) alongside methotrexate (MTX) (20 mg/m^2^ on Day +1, 15 mg/m^2^ on Days +3, +6, and +11), aimed at mitigating graft‐versus‐host disease (GVHD). In unrelated transplantation and haploidentical HSCT scenarios, alongside CsA and MTX, patients also received basiliximab (20 mg/day on Day 0 and Day +4) and 0.5 g of mycophenolate mofetil (taken orally twice daily) for additional GVHD prevention. For haploidentical transplantation, if the HLA‐matching between donor and recipient was more than 5/10, 3 mg/kg of ATG (from Day −1 to Day 0) would be given to patients. However, if the HLA‐matching between donor and recipient was 5/10, 3 mg/kg of ATG was administered for three consecutive days (from Days −3 to −1).

### 2.7 Supportive care

All patients received prophylaxis for fungal, bacterial, and viral infection according to previously published criteria.^3^, ^19^ The concentrations of Epstein–Barr virus (EBV) and cytomegalovirus (CMV) were measured weekly for 100 days after transplantation, then once every 2 weeks until 180 days after transplantation, and then once a month until 1 year after transplantation. Surveillance of bone marrow cytology and MRD status was performed once every month up to 6 months following transplantation, followed by subsequent assessments once every 3 months until the 24‐month post‐transplantation mark.

### 2.8 Statistical analysis

The process of data analysis was conducted using GraphPad Prism 8.0, SPSS 26.0, and R 3.6.3 packages. Categorical data were subjected to analysis using either the chi‐squared test or Fisher's exact test. For continuous data, statistical differences were determined using the Mann–Whitney *U*‐test. The incidence of acute GVHD and chronic GVHD, relapse, and TRM were assessed using the competing risk approach and the analysis of comparisons was performed by Fine‐Gray test. Analysis of overall survival (OS) and disease‐free disease (DFS) was conducted using the Kaplan–Meier technique, and statistical comparisons were made using log‐rank tests. The 95% confidence interval was estimated by the Brookmeyer and Crowley method. The study used a multivariate analysis approach, specifically using Cox's proportional hazard regression model to evaluate associations between potential variables and OS, DFS, and relapse. All reported *p*‐values were two‐sided, and a *p*‐value <0.05 was deemed statistically significant.

## 3 RESULTS

### 3.1 Patient characteristics

The primary patient characteristics in this study are outlined in Table 1. There were no significant variations observed among the baseline characteristics of patients between the MRD‐positive and MRD‐negative groups.

**TABLE 1:** Main characteristics in high‐risk ALL patients received allo‐HSCT.

	MRD negative	MRD positive	*p*‐value
Number of patients	90	51	
Patient Median age, years (range)	29 (6–57)	31 (13–55)	0.638
Gender			1.000
Male	52 (57.8%)	29 (56.9%)	
Female	38 (42.2%)	22 (43.1%)	
Conditioning regimen			0.860
TMLI/VP16/CY	38 (42.2%)	23 (45.1%)	
BU/VP16/CY	52 (57.8%)	28 (54.9%)	
Disease state			0.033
CR1	76 (84.4%)	35 (68.6%)	
≥CR2	14 (15.6%)	16 (31.4%)	
Immunophenotype			
B cell	71 (78.9%)	40 (78.4%)	0.980
T cell	15 (16.7%)	9 (17.7%)	
Unspecified	4 (4.4%)	2 (3.9%)	
Molecular subtype			0.975
BCR::ABL1	20 (22.2%)	15 (29.4%)	
MLL	8 (8.9%)	6 (11.7%)	
PAX5alt	9 (10.0%)	7 (13.7%)	
TCF3::PBX1	5 (5.6%)	2 (3.9%)	
IKZF1	4 (4.4%)	2 (3.9%)	
Low hypodiploid	5 (5.6%)	4 (7.8%)	
Median time from diagnosis to HSCT, month (range)	6.6 (2.9–10.4)	7.2 (3.5–11.5)	0.485
Donor/recipient (HLA)			0.810
Donor/recipient‐related HSCT			
HLA matching: 10/10	17 (18.9%)	7 (13.7%)	
HLA matching: 9/10	7 (7.8%)	3 (5.9%)	
HLA matching: 5/10 to 8/10	48 (53.3%)	28 (54.9%)	
Donor/recipient‐unrelated matched HSCT	18 (20.0%)	13 (25.5%)	
ABO match			0.727
Mismatched	44 (48.9%)	27 (52.9%)	
Matched	46 (51.1%)	24 (47.1%)	
HCT‐CI			0.676
0–1	71 (78.9%)	38 (74.5%)	
≥2	19 (21.1%)	13 (25.5%)	
Median nucleated cells, ×10^8^/kg (range)	16.13 (7.29–42.58)	17.06 (6.35–43.27)	0.253
Median CD34^+^ cells, ×10^6^/kg (range)	6.52 (3.13–15.43)	7.19 (2.49–17.76)	0.517
Median follow‐up for survivors, months (range)	28.5 (11.0–70.0)	30.7 (12.9–67.4)	0.469

Abbreviations: ALL, acute lymphoblastic leukemia; allo‐HSCT, allogeneic hematopoietic stem cell transplantation; CR, complete remission; CY, cyclophosphamide; HCT‐CI, hematopoietic cell transplantation‐specific comorbidity index.; HLA, human leukocyte antigen; MRD, minimal residual disease; Ph, Philadelphia; TMLI, total marrow and lymphoid irradiation; VP16, etoposide.

### 3.2 Engraftment and chimerism

All patients with high‐risk ALL successfully obtained hematopoietic cell engraftment after receiving intensive allo‐HSCT. No significant differences were observed in the median time of neutrophil engraftment between the two groups (MRD‐negative group: 12 days, MRD‐positive group: 11 days). Likewise, the median platelet engraftment time was also not different between the two groups (MRD‐negative group: 13 days, MRD‐positive group: 12 days). Every patient attained complete donor chimerism within 1 month after transplantation.

### 3.3 Regimen‐related toxicities and late complications

The regimen‐related toxicities are outlined in Table 2. Oral mucositis was the most common adverse effect in patients who received the TMLI‐VP16‐CY or BU‐VP16‐CY conditioning regimen. The adverse effects with the second‐highest incidence, behind mucositis, were grade 1–2 gastrointestinal toxicities. In addition to oral mucositis and nausea and diarrhea, other adverse effects, including toxicity relating to the liver, bladder, heart, kidney, central nervous systems, and lungs, were also observed in a tiny fraction of patients undergoing allo‐HSCT. The incidences of grade 3 and grade 4 regimen‐related toxicities were 16.3% and 18.0% in patients who received the BU‐VP16‐CY and TMLI‐BU‐VP16 regimens, respectively, and the difference between groups was not statistically significant. Besides, we also assessed the late complications for patients, the results demonstrated that there was no significant difference between two conditioning regimens (Table 3).

**TABLE 2:** Regimen‐related toxicity was assessed according to the Bearman's Grading System in high‐risk ALL patients receiving TMLI‐VP16‐CY or BU‐VP16‐CY conditioning regimen.

	BU‐VP16‐CY regimen				TMLI‐VP16‐CY regimen				*p‐*value
	Grade 1	Grade 2	Grade 3	Grade 4	Grade 1	Grade 2	Grade 3	Grade 4	
Oral mucositis	30 (37.5)	18 (22.5)	3 (3.8)	0 (0.0)	15 (24.6)	11 (18.0)	2 (3.3)	0 (0.0)	0.184
GI toxicities	30 (37.5)	20 (25.0)	5 (6.3)	0 (0.0)	15 (24.6)	10 (16.4)	4 (6.6)	0 (0.0)	0.075
Hepatic toxicities	6 (7.5)	4 (5.0)	1 (1.3)	1 (1.3)	4 (6.6)	2 (3.3)	1 (1.6)	0 (0.0)	0.978
Bladder toxicities	4 (5.0)	3 (3.8)	2 (2.5)	0 (0.0)	2 (3.3)	1 (1.6)	1 (1.6)	0 (0.0)	0.867
Cardiac toxicities	3 (3.8)	2 (2.5)	0 (0.0)	0 (0.0)	1 (1.6)	1 (1.6)	0 (0.0)	0 (0.0)	0.845
Kidney toxicities	2 (2.5)	1 (1.3)	0 (0.0)	0 (0.0)	4 (6.6)	1 (1.6)	0 (0.0)	0 (0.0)	0.514
CNS toxicities	1 (1.3)	1 (1.3)	1 (1.3)	0 (0.0)	0 (0.0)	1 (1.6)	0 (0.0)	0 (0.0)	1
Pulmonary toxicities	1 (1.3)	1 (1.3)	0 (0.0)	0 (0.0)	1 (1.6)	0 (0.0)	0 (0.0)	0 (0.0)	1

*Note*: Data are no. of patients (%).

Abbreviations: ALL, acute lymphoblastic leukemia; BU, busulfan; CNS, central nervous system; CY, cyclophosphamide; TMLI, total marrow and lymphoid irradiation; VP16, etoposide.

**TABLE 3:** Late complications in high‐risk ALL patients receiving TMLI‐VP16‐CY or BU‐VP16‐CY conditioning regimen.

	BU‐VP16‐CY regimen	TMLI‐VP16‐CY regimen	*p‐*value
Cardiovascular disease	3 (3.8)	4 (6.6)	0.466
Endocrine and Metabolic Disorders	7 (8.8)	6 (9.8)	1
Pulmonary complications	2 (2.5)	5 (8.2)	0.403
Liver complications	3 (3.8)	3 (4.9)	1
Renal complications	2 (2.5)	1 (1.6)	1
Eye complication	5 (6.3)	3 (4.9)	1
Venous thromboembolism	4 (5.0)	2 (3.3)	0.698
Infertility	14 (17.5)	10 (16.4)	1
Bone and joint complications	5 (6.3)	5 (8.2)	1
Secondary solid tumors	0	0	—

*Note*: Data are no. of patients (%).

Abbreviations: ALL, acute lymphoblastic leukemia; BU, busulfan; CY, cyclophosphamide; TMLI, total marrow and lymphoid irradiation; VP16, etoposide.

### 3.4 Acute and chronic GVHD

**FIGURE 1:**
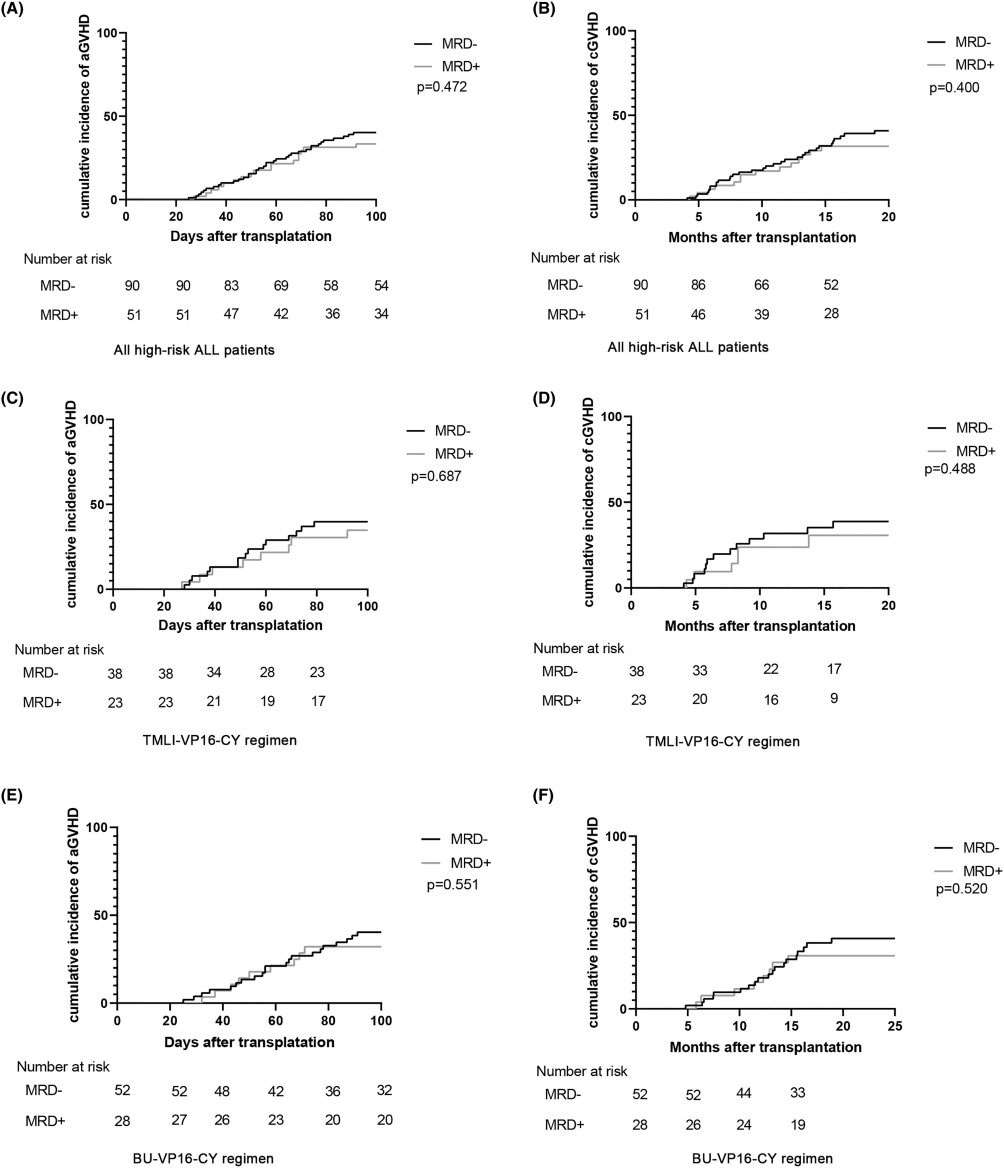
Incidence of aGVHD and cGVHD in patients who underwent allo‐HSCT for high‐risk acute lymphoblastic leukemia (ALL). The incidence of aGVHD and cGVHD among all high‐risk ALL patients who underwent allo‐HSCT with TMLI‐VP16‐CY and BU‐VP16‐CY conditioning regimens (A and B). Panels (C) and (D) represent the incidence of aGVHD and cGVHD for high‐risk ALL patients subjected to the TMLI‐VP16‐CY regimen, while panels (E) and (F) illustrate incidence of aGVHD and cGVHD for those who received the BU‐VP16‐CY regimen.

The occurrence of acute and chronic GVHD significantly influences patient outcomes following allo‐HSCT. Therefore, we investigated the incidence of GVHD within the two groups. Among the high‐risk ALL patients subjected to intensified allo‐HSCT, no substantial differences emerged in the incidence of aGVHD and cGVHD between the MRD‐negative and MRD‐positive groups. The cumulative incidence of grade I to IV aGVHD at 100 days was 40.2% (39.6%–40.7%) and 33.4% (32.5%–34.3%) for the MRD‐negative and MRD‐positive groups, respectively (*p* = 0.472) (Figure 1A). Correspondingly, the 100‐day incidence of grade II to IV aGVHD for these two groups was 26.7%, and 21.8%, respectively (*p* = 0.371). Furthermore, there was no statistically significant difference in the 100‐day incidence of grade III to IV acute graft‐versus‐host disease (aGVHD) between the two groups (MRD‐negative group: 14.5%; MRD‐positive group: 15.1%; *p* = 0.419). The cumulative incidence of cGVHD at 2 years was also unchanged between the two groups (MRD‐negative group: 40.9% (40.2%–41.5%); MRD‐positive group: 31.6% (30.7%–32.7%); *p* = 0.400) (Figure 1B). Additionally, the choice of conditioning regimen did not appear to influence GVHD incidence, as aGVHD and cGVHD incidences were similar between patients given the TMLI‐VP16‐CY regimen and those treated with the BU‐VP16‐CY regimen (Figure 1C–F).

### 3.5 Relapse

**FIGURE 2:**
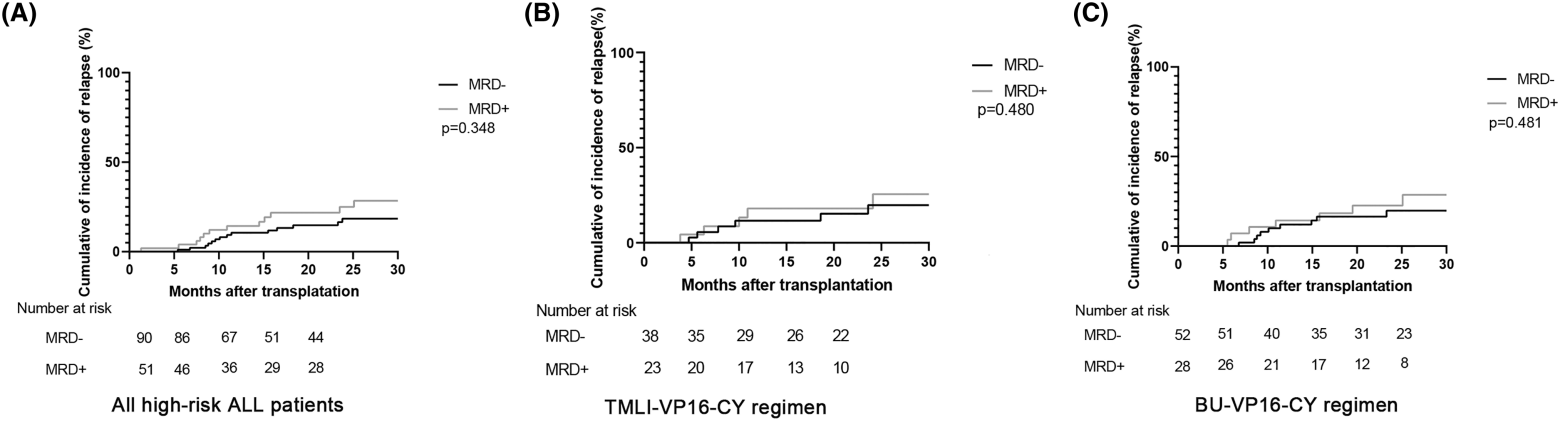
Incidence of relapse among patients who received allo‐HSCT for high‐risk acute lymphoblastic leukemia (ALL). The incidence of relapse for all high‐risk ALL patients undergoing allo‐HSCT with TMLI‐VP16‐CY and BU‐VP16‐CY conditioning regimens (A). Panel (B) portrays the incidence of relapse for high‐risk ALL patients receiving the TMLI‐VP16‐CY regimen, while panel (C) represents the incidence of relapse for those treated with the BU‐VP16‐CY regimen. The notation “ns” indicates no statistical significance.

Due to the significance of MRD in contributing to relapse among allo‐HSCT patients, we further sought to discern whether the addition of VP16 has the potential to reduce the overall rate of relapse in high‐risk patients with ALL who tested positive for MRD. Our findings demonstrated that the projected 2‐year relapse rate was not higher in MRD‐positive patients compared with MRD‐negative patients (32.2% (31.2%–33.2%) vs. 25.0% (24.8%–25.5%), *p* = 0.348) (Figure 2A). Specifically, in the MRD‐negative remission group, eight patients had hematologic relapse, four patients had cytogenetic relapse, and four had extramedullary relapse. In the MRD‐positive group, seven patients had a relapse after transplantation, including three patients with hematologic relapse, two patients with cytogenetic relapse, and two patients with extramedullary relapse. In this study, the vast majority of patients did not receive post‐transplant maintenance therapy, and only 4 Ph^+^ALL patients who were MRD‐positive before transplantation received TKI therapy after transplantation for maintenance.

Among patients who received the TMLI‐VP16‐CY regimen, no notable increase in the cumulative rate of relapse was observed in the MRD‐positive group compared with the MRD‐negative cohort (28.6% (25.95–31.2%) vs. 20.4% (19.2%–21.6%), *p* = 0.480) (Figure 2B). Similar outcomes were also observed in both groups undergoing the BU‐VP16‐CY regimen (Figure 2C). These data indicate that the TMLI‐VP16‐CY and BU‐VP16‐CY conditioning regimens could effectively control disease relapse in high‐risk ALL patients receiving allo‐HSCT who were MRD‐positive prior to transplantation.

### 3.6 Transplantation‐related mortality

**FIGURE 3:**
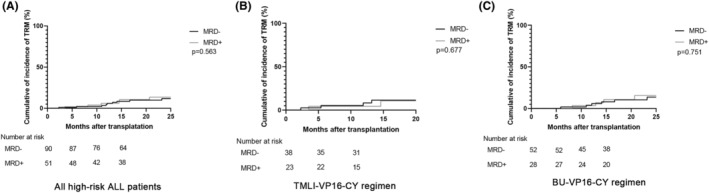
Incidence of transplantation‐related mortality (TRM) among patients who underwent allo‐HSCT for high‐risk acute lymphoblastic leukemia (ALL). The incidence of TRM for all high‐risk ALL patients undergoing allo‐HSCT with TMLI‐VP16‐CY and BU‐VP16‐CY conditioning regimens (A). Panel (B) portrays the incidence of TRM for high‐risk ALL patients receiving the TMLI‐VP16‐CY regimen, while panel (C) represents the incidence of TRM for those treated with the BU‐VP16‐CY regimen.

Transplantation‐related mortality (TRM) is another important factor affecting the efficacy of transplantation. Therefore, we also assessed the 1‐year estimated incidence of TRM among high‐risk ALL patients. The findings indicated that there was no remarkable difference in the incidence of TRM between the MRD‐negative cohort and the MRD‐positive cohort (Figure 3A–C). In detail, the causes leading to transplantation‐related mortality were as follows: within the MRD‐negative group, three patients died of grade IV intestinal aGVHD, one patient died of grade IV hepatic aGVHD, one patient died of bronchiolitis obliterans (cGVHD), one patient developed renal cGVHD and eventually died of chronic renal failure, one patient died of pulmonary *Aspergillus* infection, and another died of *Pseudomonas aeruginosa* lung infection. In addition, one patient died of hemorrhagic cystitis. In the MRD‐positive group, three patients died of grade IV aGVHD, one patient died of bronchiolitis obliterans (cGVHD), one patient died of bloodstream infection with *Klebsiella pneumoniae*, and one patient died from thrombotic microangiopathy (Table 4).

**TABLE 4:** Causes of high‐risk ALL patients underwent allo‐HSCT.

	MRD negative	MRD positive	*p*‐value
Number of patients, *n* (%)	90	51	
Relapse, *n* (%)	8 (8.9)	7 (13.7)	0.403
TRM, *n* (%)	9 (10.0)	6 (11.8)	0.780
aGVHD, *n* (%)	4 (4.4)	3 (5.8)	
cGVHD, *n* (%)	2 (2.2)	1 (2.0)	
Infection, *n* (%)	2 (2.2)	1 (2.0)	
Other, *n* (%)	1 (1.1)	1 (2.0)	

*Note*: Data are no. of patients (%).

Abbreviations: aGVHD, acute graft‐versus‐host disease; ALL, acute lymphoblastic leukemia; cGVHD, chronic graft‐versus‐host disease; MRD, minimal residual disease; TRM, transplantation‐related mortality.

### 3.7 OS and DFS

**FIGURE 4:**
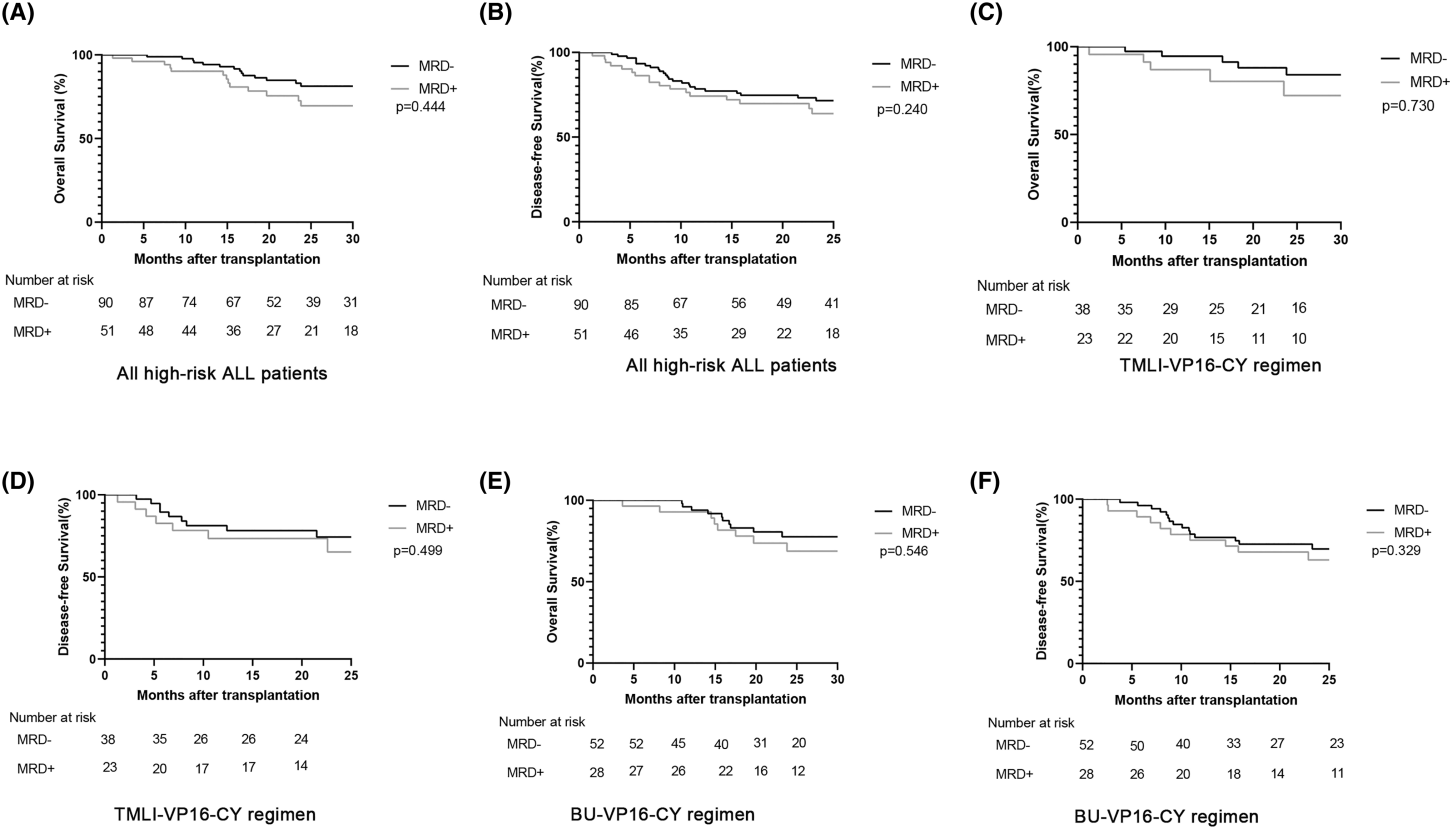
Overall survival (OS) and disease‐free survival (DFS) outcomes among patients who underwent allo‐HSCT for high‐risk acute lymphoblastic leukemia (ALL). The OS and DFS outcomes for all high‐risk ALL patients who underwent allo‐HSCT with TMLI‐VP16‐CY and BU‐VP16‐CY conditioning regimens (panels A and B). Panels (C) and (D) illustrate the OS and DFS for high‐risk ALL patients receiving the TMLI‐VP16‐CY regimen, while panels (E) and (F) represent the OS and DFS for those treated with the BU‐VP16‐CY regimen.

**FIGURE 5:**
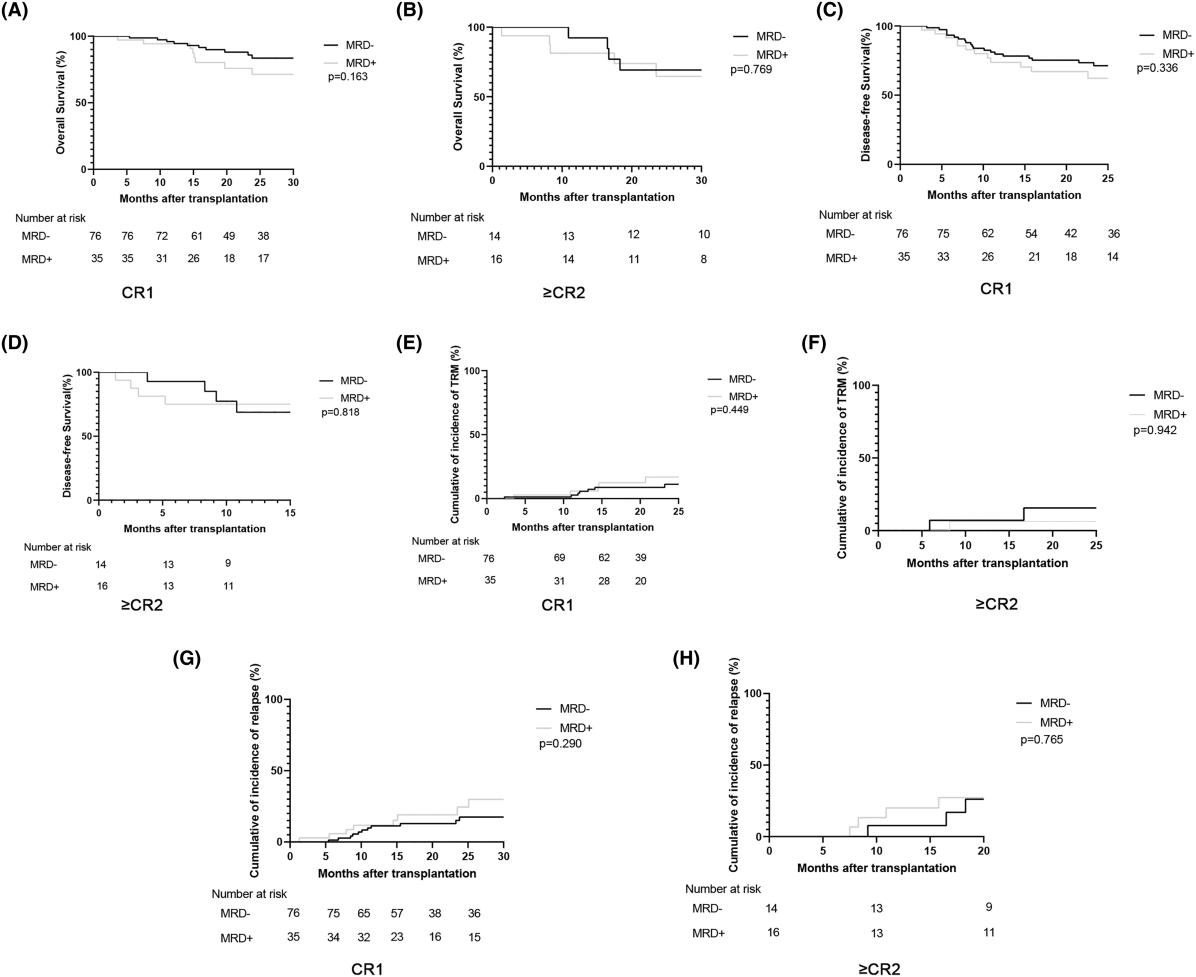
Outcomes of acute lymphoblastic leukemia (ALL) patients received transplantation in CR1 and ≥CR2. The overall survival (A, B) and disease‐free survival (C, D) outcomes for ALL patients who were in CR1 and ≥CR2. The cumulative incidence of transplantation‐related mortality (E, F) and relapse (G, H) for ALL patients who were in CR1 and ≥CR2.

In the cohort of high‐risk ALL patients who received allo‐HSCT, there was no statistical difference in the projected 2‐year OS (69% (61.9%–82.2%) vs. 66.7% (53.9%–82.5%), *p* = 0.444) nor the 2‐year DFS (67.2% (57.9%–78.1%) vs. 55.5% (42.6%–72.3%), *p* = 0.240) between the MRD‐negative group and the MRD‐positive groups (Figure 4A,B). In patients who received the TMLI‐VP16‐CY conditioning regimen, the estimated 2‐year OS (74.2% (60.8%–90.6%) vs. 70.1% (52.1%–94.4%), *p* = 0.730) (Figure 4C) and 2‐year DFS (73.0% (59.9%–88.8%) vs. 67.8% (50.4%–91.2%), *p* = 0.499) (Figure 4D) were similar in both the MRD‐negative and MRD‐positive groups. Similarly, among patients given the BU‐VP16‐CY regimen, no noteworthy difference was observed in the projected OS and 2‐year DFS between the MRD‐negative and MRD‐positive groups (Figure 4E,F). Additionally, we also separately assessed survival analysis in the CR1 and ≥CR2 subgroups; the results showed that there was no significant difference in OS, relapse, DFS, or TRM between MRD‐positive and MRD‐negative patients regardless of whether they were classified as ≥CR2 or CR1 (Figure 5A–H). These data indicate that both the TMLI‐VP16‐CY and BU‐VP16‐CY conditioning regimens could improve OS and DFS among MRD‐positive allo‐HSCT patients, to achieve outcomes similar to those of MRD‐negative patients.

### 3.8 Multivariate analysis

To determine the specific factors associated with OS, DFS, and relapse, univariate analysis and multivariate analysis were performed. Valuable variables from the univariate analysis were included in the multivariate analysis. The findings indicated that these variables including haploidentical transplantation, cGVHD and the selected conditioning regimen were not significantly associated with DFS, or relapse among high‐risk ALL patients subjected to allo‐HSCT. However, cGVHD was significantly associated with OS (Table 5).

**TABLE 5:** Multivariable analysis for relapse, OS, and DFS in high‐risk ALL patients received allo‐HSCT.

	Univariate analysis	*p*‐value	Multivariate analysis	*p*‐value
	Hazard ratio (95% CI)		Hazard ratio (95% CI)	
Relapse				
Haploidentical vs. nonhaploidentical	0.558 (0.230–1.354)	0.197	0.557 (0.228–1.357)	0.198
cGVHD vs. no cGVHD	1.393 (0.539–3.597)	0.494	1.425 (0.548–3.705)	0.468
Conditioning regimen (TMLI/VP16/CY vs. BU/VP16/CY)	1.275 (0.533–3.048)	0.585	1.233 (0.512–2.971)	0.641
OS				
Haploidentical vs. nonhaploidentical	0.764 (0.326–1.789)	0.535	0.747 (0.313–1.783)	0.511
cGVHD vs. no cGVHD	3.354 (1.086–10.362)	0.035	3.403 (1.098–10.548)	0.034
Conditioning regimen (TMLI/VP16/CY vs. BU/VP16/CY)	1.376 (0.580–3.265)	0.469	1.356 (0.561–3.277)	0.498
DFS				
Haploidentical vs. nonhaploidentical	0.577 (0.273–1.217)	0.149	0.567 (0.265–1.212)	0.143
cGVHD vs. no cGVHD	2.088 (0.898–4.854)	0.087	2.157 (0.919–5.061)	0.077
Conditioning regimen (TMLI/VP16/CY vs. BU/VP16/CY)	1.477 (0.699–3.117)	0.307	1.439 (0.672–3.082)	0.349

Abbreviations: ALL, acute lymphoblastic leukemia; Bu, Busulfan; cGVHD, chronic graft‐versus‐host disease; CI, confidence interval; CR, complete remission; CY, cyclophosphamide; DFS, disease‐free survival; OS, overall survival; TMLI, total marrow and lymphoid irradiation; VP16, etoposide.

## 4 DISCUSSION

HSCT is an important treatment for ALL, especially for high‐risk ALL.^2^, ^23^ Unfortunately, although considerable advancements have been achieved in this domain, post‐transplant relapse remains a crucial contributor to graft failure. Several studies have shown that the disease status of the patient prior to transplantation is a risk factor for relapse post‐transplant.^20^, ^24^ Individuals with ALL who successfully attain a state of CR in the bone marrow but exhibit MRD positivity prior to transplantation are at an elevated risk of experiencing relapse following the transplantation procedure,^20^ which is a major clinical challenge. As the pre‐transplant conditioning regimen is a key factor affecting the efficacy of transplantation, it is important to determine the optimal conditioning regimen for improving the prognosis of MRD‐positive ALL patients. In this study, we found that both intensified TMLI‐VP16‐16CY and BU‐VP16‐CY could decrease the incidence of relapse among MRD‐positive patients and increase the OS and DFS, a result that was on par with that of MRD‐negative patients undergoing transplantation.

MRD assessment is used to identify low levels of leukemic cells^7^ and to provide guidance for subsequent chemotherapy or transplantation options. Although it would be ideal to achieve CR and MRD‐negative status before transplantation, some individuals fail to progress from MRD‐positive to MRD‐negative status, even after undergoing additional chemotherapy.^25^ Transplantation may still be performed despite MRD positivity based on a complex plethora of factors relating to the patient, including limitations in tolerance to chemotherapy, the possibility of chemotherapy‐induced drug resistance in the leukemia cells, and the ideal window for the time of transplantation. Despite transplantation being a viable choice in most cases, one study reported an elevated incidence of relapse among MRD‐positive patients compared with those with MRD‐negative status.^21^ Moreover, another study reported that the 5‐year DFS in MRD‐positive ALL patients was only 29.9%.^26^ Therefore, the prognosis of MRD‐positive patients is poor. Significance research efforts have focused on potential strategies to further reduce the relapse rate of ALL patients after transplantation. One option is to use more intensive conditioning regimens to achieve improved efficacy in eliminating leukemia cells,^23^, ^27^, ^28^ thereby benefiting patients to a greater extent. Therefore, for MRD‐positive patients with high‐risk ALL, it may be beneficial to implement an intensive conditioning regimen to effectively clear the leukemia cells prior to hematopoietic stem cell infusion to reduce the recurrence rate. VP16 induces cell cycle arrest, apoptosis, and autophagy within tumor cells.^29^, ^30^ In our study, we observed that VP‐16‐intensified TMLI‐CY and BU‐CY regimens seemed to be able to eliminate leukemia cells more thoroughly. Indeed, the data demonstrates a decrease in relapse occurrence among individuals with MRD‐positive status, such that their outcomes were equivalent to those in MRD‐negative patients.

Although relapse is an important factor in transplant failure, regimen‐related toxicity after transplantation also needs to be considered. The preparatory regimen involving TBI is widely used in ALL patients before transplantation.^31^ Although it significantly reduces the relapse rate of ALL patients to a certain extent, the long‐term and short‐term radiotherapy‐related toxicity caused by TBI also largely limits the efficacy of transplantation in ALL patients.^12^ Therefore, the appropriate conditioning regimen should prevent relapse without increasing regimen‐related toxicity, requiring clinicians to find the optimal balance between intensity and tolerability to maximize the benefits for individuals who are in need of hematopoietic stem cell transplantation. Fortunately, the emergence of TMLI‐based^32^ and BU‐based regimens^31^, ^33^ has enabled more options for ALL patients. Trial results have shown that TMLI reduced both recurrence rate and radiotherapy‐related toxicity.^34^ Similarly, the BU‐based protocol also achieved comparable OS to TBI.^33^ Therefore, in this study, we attempted to replace TBI with TMLI and BU as part of the pretreatment protocol. Due to the potent mucosal toxicity and cardiotoxicity of IDA, we switched to VP16 as part of the intensive conditioning protocol for patients with MRD‐positive ALL undergoing transplantation, with the objective of reducing the incidence of relapse without increasing serious treatment‐related toxicity. Indeed, the results of the present study indicated that the regimen‐related toxicities were acceptable. The most common adverse effects were oral mucositis and nausea and diarrhea, all of which improved with intervention.

We observed no notable disparity in relapse rates between MRD‐positive and MRD‐negative patients with either the TMLI‐VP16‐CY or BU‐VP16‐CY conditioning regimen, and no substantial differences in terms of regimen‐related toxicity or transplant‐related mortality. Furthermore, both the MRD‐positive patients and MRD‐negative patients who received VP16‐intensified conditioning showed similar 2‐year DFS and 2‐year OS. These results suggest that VP16‐intensified TMLI‐CY and BU‐CY conditioning regimens are beneficial for MRD‐positive ALL patients, who achieved outcomes comparable to those in MRD‐negative patients. Based on the above findings, our regimen achieved our expected goal. Therefore, more work is needed in the future to further optimize the regimen to prolong the survival of individuals who have an ongoing medical condition at the time of receiving an organ transplant. For example, in addition to conditioning regimens, post‐transplantation medical therapy may be administered to high‐risk patients to prevent relapse. Currently, some studies have shown that TKI maintenance therapy should be recommended for Ph^+^ALL patients after transplantation to prevent relapse in high‐risk ALL patients.^35^, ^36^ Additionally, some innovative strategies are also being developed. One clinical trial indicated that blinatumomab was feasible as maintenance therapy in patients with high‐risk B‐lineage ALL after transplantation.^37^ However, data on post‐transplantation maintenance therapy is relatively scarce for T‐ALL compared with B‐ALL. Nevertheless, one study showed that four patients with high‐risk T‐ALL who received 5‐azacitidine and venetoclax as maintenance therapy after transplantation achieved sustained complete remission, and tolerated this treatment for a median follow‐up period of 15 months.^38^ These studies will pave the way for the maintenance treatment of patients after transplantation. In addition to maintenance therapy, the donor source, and GVHD prophylaxis are also important for the outcomes of ALL patients who underwent transplantation. But in our study, no difference in relapse, OS, and DFS between haploidentical and nonhaploidentical transplantation was observed, which was similar to this study.^39^ Besides, one research showed that a reduction in GVHD intensity to increase GVL effects in Ph^+^ALL patients is not recommended.^40^ However, there are no relevant data on whether different GVHD prevention regimens can overcome the poor post‐transplantation outcomes caused by positive MRD before transplantation, which is also a research direction in the future.

We recognize some limitations in our present study. First, a constraint of this study is its retrospective nature, encompassing a limited sample size that could potentially introduce biases. Therefore, it is crucial that future research endeavors include randomized controlled trials with larger sample sizes to validate our results. Second, because the BU concentration was not monitored and dose modification was not performed in this study, we did not evaluate the relationship between BU concentration and efficacy in ALL patients undergoing HSCT. In our future research, we intend to actively monitor and adjust the BU dosing.

In conclusion, TMLI‐VP16‐CY and BU‐VP16‐CY preparatory regimens exhibit effectiveness in managing high‐risk ALL patients with MRD‐positive status and CR. Both the TMLI‐VP16‐CY and BU‐VP16‐CY intensive conditioning regimens could reverse the possible poor prognosis caused by MRD positivity at transplantation, substantially decrease the incidence of relapse and enhance the overall survival and DFS outcomes for MRD‐positive patients following transplantation. Moreover, the regimen‐related toxicities and TRM were acceptable. Notably, these regimens could achieve similar transplantation outcomes in MRD‐positive patients as those in MRD‐negative patients. Together, our findings unequivocally underscore the efficacy of the TMLI‐VP16‐CY and BU‐VP16‐CY conditioning regimens in managing high‐risk patients with MRD‐positive disease. These results have important implications for guiding clinical decisions in the near future.

## AUTHOR CONTRIBUTIONS

**Xiaoyan Zhao:** Data curation (equal); formal analysis (equal); methodology (equal); writing – original draft (lead). **Ziwei Xu:** Data curation (equal); methodology (lead); resources (equal). **Ziying Li:** Investigation (equal); resources (equal); visualization (equal). **Xi Zhou:** Resources (equal); software (equal). **Yu Hu:** Conceptualization (lead); supervision (equal); writing – review and editing (lead). **Huafang Wang:** Conceptualization (lead); formal analysis (lead); funding acquisition (equal); project administration (lead); supervision (lead).

## ETHICS STATEMENT

This investigation adhered to the guidelines outlined in the Declaration of Helsinki. Ethical endorsement for this study was granted by the Medical Ethics Committee at Huazhong University of Science and Technology. Comprehensive informed consent was procured from all participants who were enrolled in the research.

## Data Availability

The data for the findings of this research can be requested from the corresponding author upon reasonable request.
